# Isolation, Characterization and Antioxidant Activity of Yam Polysaccharides

**DOI:** 10.3390/foods11060800

**Published:** 2022-03-10

**Authors:** Zhedong Li, Wenhao Xiao, Jianhua Xie, Yi Chen, Qiang Yu, Weidong Zhang, Mingyue Shen

**Affiliations:** State Key Laboratory of Food Science and Technology, Nanchang University, Nanchang 330047, China; 15639052109@163.com (Z.L.); xwh970726@163.com (W.X.); jhxie@ncu.edu.cn (J.X.); chenyi15@ncu.edu.cn (Y.C.); yuqiang8612@163.com (Q.Y.); 15502618197@163.com (W.Z.)

**Keywords:** Chinese yam, polysaccharide, structural characterization, oxidative stress, antioxidant activity

## Abstract

This study aimed to characterize the structure of Chinese yam (*Dioscoreae Rhizoma*) polysaccharide (CYP) and to investigate its protective effect against H_2_O_2_-induced oxidative damage in IEC-6 cells. The chemical composition and structural characteristics of the samples were analyzed by chemical and instrumental methods, including high-performance gel permeation chromatography, high-performance anion-exchange chromatography (HPAEC), Fourier transformed infrared (FT-IR), ultraviolet (UV), and scanning electron microscopy (SEM). Antioxidant activity was evaluated by establishing a cellular model of oxidative damage. The molecular weight of CYP was 20.89 kDa. Analysis of the monosaccharide composition revealed that CYP was primarily comprised of galactose (Gal), glucose (Glu), and galacturonic acid (GalA), and the ratio between them was 28.57:11.28:37.59. Pretreatment with CYP was able to improve cell viability, superoxide dismutase (SOD) activity, and reduce intracellular reactive oxygen species (ROS) production and malondialdehyde (MDA) content after H_2_O_2_ injury. CYP also attenuated oxidative damage in cells through the mitogen-activated protein kinase (MAPK) signaling pathway. This study showed that CYP was an acidic heteropolysaccharide with a good protective effect against oxidative damage, and it thus has good prospects in food and biopharmaceutical industries.

## 1. Introduction

Cellular and tissue oxidative stress is a response to stimulation by increased reactive oxygen species (ROS) and free radicals in the body, resulting in dysregulation of the intracellular oxidative antioxidant system [1]. Cells can catalyze the superoxide anion produced in oxidative stress into H_2_O_2_, which is broken down into water and oxygen in one step by inducing superoxide dismutase (SOD), catalase, and glutathione peroxidase [2,3]. Oxidative stress could also disrupt intestinal homeostasis, which is a critical element in the development of intestinal damage. Stress can induce intestinal cells to produce large amounts of ROS metabolites, which affect the stability of intracellular nucleic acids, proteins, and lipids, increase apoptosis, inflict intestinal mucosal damage, and induce inflammatory bowel disease [4,5]. Many small intestinal epithelial cells exist on the surface of the intestine, and as a mediator of the internal and external environment of the intestine, the small intestinal epithelial cells are also part of the immune barrier that can defend the body against pathogens and other harmful substances [6,7,8,9]. Studies have shown that polysaccharides can scavenge ROS and enhance the antioxidant system to improve intestinal inflammation, therefore, it is relevant to find an antioxidant to protect the intestine from oxidative damage [10,11,12,13].

Yam is a plant from the *Dioscoreaceae* family. It grows in warm, low-altitude environments and is distributed throughout Asia, primarily in Korea, Japan, and China [14]. It contains polysaccharides, amino acids, fatty acids, trace elements, starch, protein, and other components [15]. Polysaccharides extracted from yam have a variety of activities such as antioxidant, antitumor, immunomodulatory, and hypoglycemic. The monosaccharide composition of yam polysaccharides is complicated and caused by factors such as the growth conditions and different extraction methods. However, ribose is not found in the monosaccharide composition of most yam polysaccharides. Polysaccharides obtained from Chinese yams generally show good antioxidant activity in vitro, whereas glucose accounts for a large proportion of their monosaccharide composition. Based on the report of Liu et al., the molar ratios of glucose in the polysaccharides they obtained from Chinese yam all exceed 80% [16,17,18,19,20].

Over the past decade, antioxidant studies on yam polysaccharides have primarily focused on the free-radical scavenging ability in vitro. According to Zhu et al. [21], CYP has a strong antioxidant capacity and could effectively scavenge DPPH, ABTS+ and ·OH radicals, especially for DPPH radicals, whose scavenging ability is comparable to that of ascorbic acid (Vc). However, few studies have been conducted on the antioxidant activity of CYP at the cellular level. Despite salvia glycoproteins can increase the activity of antioxidant enzymes and reduce malondialdehyde (MDA) levels, the potential mechanism remains unknown. Therefore, it is meaningful to study this mechanism at the cellular level [22].

Despite the multiple biological activities of CYP, its structural features remain unclear and incomplete [23,24,25]. The structures of yams grown in different environments at different times are not quite the same, and their biological activities might still be different [26]. In the present study, we established a model of oxidative loss damage of cells by H_2_O_2_ and explored the protective effects of CYP on the oxidative damage of IEC-6 through cell-viability experiments and the levels of SOD and MDA levels.

## 2. Materials and Methods

### 2.1. Materials and Reagents

Fresh yam (*Dioscoreae Rhizoma*), which was plucked in November and purchased from Jiujiang, Jiangxi, China, was powdered with a high-speed disintegrator (XL-20B, Zhengzhou Fengli Crushing Equipment Co., Ltd., Zhengzhou, China). Fetal bovine serum (FBS) and Dulbecco’s modified eagle medium (DMEM) were purchased from Solarbio Science and Technology Co., Ltd. (Beijing, China). The cell counting kit-8 (CCK-8) was purchased from Dojindo Molecular Technologies, Inc. (Kyushu Island, Japan). The reactive oxygen species kit, superoxidase dismutase kit, and malondialdehyde kit were purchased from Beyotime Biotechnology (Shanghai, China). Hydrogen peroxide (H_2_O_2_) was purchased from Sigma (Sigma, Burlington, MA, USA). The IEC-6 cell line was purchased from the Cell Bank of the Chinese Academy of Sciences (Shanghai, China). Antibodies used in this study were purchased from Cell Signalingas (Bever, MA, USA). All the other chemicals used in the present study were analytical reagent grade.

### 2.2. Extraction and Purification of Polysaccharides

The yam was cleaned and dried for 12 h and then peeled and sliced the next day. The sliced yam (thickness = 3 mm) was placed in a tray, dried in an oven (VYJG-9420, Hangzhou Yijie Technology Co., Hangzhou, China) at 55 °C for 48 h, taken out and ground into powder (60 mesh) with a high-speed disintegrator, after soaking in ethanol (95%) overnight to remove fat, color, and small molecules, the precipitate was collected the next day and heated in a water bath in a ventilated area to remove the ethanol and dry completely. The obtained powder was placed in a self-sealing bag and prepared for later experiments. A total of 300 g of yam powder was weighed, and 6 L of ultrapure water was added at a ratio of 1:20. After extraction at 80 °C for 3 h, the above process was repeated twice, and the extracts were combined, centrifuged, and concentrated to 1/10 of the original volume. After adding 95% ethanol (concentrate:ethanol = 1:5.33) overnight precipitate at 4 °C. The next day, the precipitate was taken and re-solubilized, then the solution pH was adjusted to 4.5 with hydrochloric acid, and glycosylase was added at 55 °C for 1 h, followed by pH adjustment to 6.5 with sodium hydroxide, amylase was added at 95 °C for 2 h, finally papain was added at 65 °C for 1.5 h, high temperature (100 °C) inactivated for 30 min, and proteins were removed 3 times by Sevage method [27], (polysaccharide liquid:chloroform:n-Butyl alcohol = 25:4:1). Then the solution was centrifuged, and the upper layer was gathered to obtain the polysaccharide solution with proteins removed, and then the solution was subjected to dialysis (Mw:8000, tap water for 24 h, distilled water for 24 h, ultrapure water for 24 h) to remove minor molecules and the inorganic ions. The yam polysaccharide solution was concentrated under reduced pressure with a vacuum pump, and four times the volume of anhydrous alcohol was added for 24 h. After centrifugation, the precipitate was placed in a lyophilizer to obtain refined yam polysaccharide.

### 2.3. Structure Characterization

#### 2.3.1. Determination of the Content of Total Carbohydrate, Uronic Acid and Protein

Carbohydrate content was determined by phenol sulfate method according to the method of Huang et al. [28]. In short, prepare 0.1 mg/mL standard glucose solution was prepared, 0.1, 0.2, 0.4, 0.6, 0.8 and 1.0 mL was pipetted in a test tube, and then add distilled water to 1 mL, after that, 1 mL of 3% phenol was added, gradually add 4 mL of concentrated sulfuric acid, shake while adding, measuring the absorbance at 490 nm after reaction for 30 min at room temperature.

Content of the uronic acid was determined by Carbazole-sulfate method [29]. In brief, galacturonic acid standard solution was configured in the same way, then 6 mL of superior pure sulfuric acid was added in an ice bath, shaking while adding, water bath at 85 °C for 25 min, taken out and cooled to room temperature, 0.2 mL of 0.1% carbazole-ethanol (25 mg carbazole dissolved in 25 mL ethanol) was added, and the reaction was carried out for 2 h. The absorbance was measured at 530 nm.

Content of protein was measured with Coomassie brilliant blue method [30]. Configure 0.1 mg/mL of BSA standard solution, and Komas brilliant blue solution (100 mg of Komas brilliant blue dissolved in 50 mL of 90% ethanol, add 100 mL of 85% phosphoric acid, distilled water volume to 1000 mL), 5 mL of Komas brilliant blue was added to the standard solution and CYP solution, plug the cap and invert 3–5 times, measure the absorbance at 595 nm.

#### 2.3.2. Determination of Molecular Weight (Mw)

Molecular weight (Mw) was evaluated on a high-performance liquid chromatography system with an ultraviolet absorption and refractive index detector.

The chromatographic column of UltrahydrogelTM-1000 was eluted with ultrapure water at a flow rate of 0.6 mL/min. The column and RI detector were maintained at 35 °C. Glucose and dextrans (T-10, T-25, T-40, T-50, T-70, and T-500) were used as standards. We prepared 1 mg/mL CYP solution and added the polysaccharide solution to the injection vial by using a syringe combined with a 0.22 μm aqueous-phase needle filter for determination.

#### 2.3.3. Monosaccharide Composition

The monosaccharide composition of CYP was determined based on the method of Xu et al. [31] with few changes. CYP (5 ± 0.05 mg) was put into a tube, and 0.5 mL of 12 M H_2_SO_4_ was added in an ice bath with magnetic stirring for 0.5 h to dissolve it completely. After 2 mL of ultrapure water was added, tubes were placed in an oil bath at 105 °C for 4 h. After heating, polysaccharide solution was fixed the volume with a 250 mL volume volumetric flask, 1 mL of sample was taken out and was filtered through 0.22 μm membrane for HPAEC analysis. Water and sodium hydroxide were used as the eluent and at a flow rate of 0.25 mL/min.

#### 2.3.4. Scanning Electron Microscope (SEM) Analysis

CYP micromorphology was observed by SEM. In a typical procedure, polysaccharide samples were placed on a conductive plate and held in place. After CYP was sprayed with gold powder, SEM was used to record images at different magnifications (500–2000 times) at an acceleration voltage of 5 kV [32].

#### 2.3.5. Spectra Analysis

The UV spectrum of CYP (0.5 mg/mL) was obtained on a spectrophotometer (TU-1900, Pgenenal, Beijing, China) within the scanning range of 190–400 nm. The FT-IR spectrum of CYP was measured with a Nicolet 5700 FT-IR spectrometer (Thermo Electron, Madison, WI, USA) within the 4000–400 cm^−1^ wavenumber region [33].

### 2.4. Cytoprotective Activity

#### 2.4.1. Cell Viability Assay

In this study, a Cell Counting Kit-8 (CCK8) kit was conducted to assess the viability of the cells. IEC-6 cells were seeded into a 96-well plate, 10^4^ cells/well. After incubation in 5% CO_2_ and 37 °C for 24 h, CYP (200,400, and 800 μg/mL) was inserted into the well for co-culturing 24 h, CCK-8 solution was added and incubated with IEC-6 cells for 30 min, followed by measuring the absorbance at 450 nm using a microplate reader.

Different concentrations (100–500 μM) of H_2_O_2_ were used to stimulate IEC-6 cells for 4 h in 96-well plates, respectively. After adding CCK-8 solution for 4 h, the absorbance was measured at 450 nm to screen the damage concentration of H_2_O_2_.

After incubating IEC-6 cells in 5% CO_2_ at 37 °C for 24 h in 96-well plates, CYP at 200, 400, and 800 μg/mL was incubated in the wells together for 24 h, respectively. IEC-6 cells were stimulated with H_2_O_2_ for 4 h, and then CCK-8 assay was performed.

#### 2.4.2. Measurement of SOD and MDA Levels

The cells (2 × 10^5^ cells/well) were seeded onto a six-well plate and incubated for 24 h to adhere to the bottom of the well plates. After CYP (200, 400, and 800 μg/mL, respectively) were added for co-culturing 24 h, cells were exposed to a final concentration of 300 µM of H_2_O_2_ for 4 h. Finally, the content of MDA and SOD contents were measured according to the manufacturer’s instructions of the assay kits.

#### 2.4.3. Measurement of Intracellular ROS Level

Intracellular ROS was tested by using 2,7-dichlorofuorescin diacetate (DCFH-DA). IEC-6 cells (2 × 10^5^ cells/well, 2 mL/well) were seeded onto a six-well plate. After incubation for 24 h, cells were exposed to H_2_O_2_ (300 μM) for 4 h before different concentrations of CYP pre-treated cells for 4 h. Fluorescent dye was added to bind to the cells for 30 min, then washed with PBS and 1640 medium, respectively. Finally, the fluorescence intensity was detected by fluorescence spectrophotometry.

#### 2.4.4. Western Blot Analysis

Aiming to further evaluate the expression of proteins in the mitogen-activated protein kinase (MAPK) pathway associated with cellular oxidative stress, pre-treat IEC-6 cells with different concentrations of CYP, after adding H_2_O_2_ to stimulate for 4 h, the cells were washed twice with PBS and then lysed the cells with RIPA buffer. After centrifugation, the samples buffer was added to the supernatant and heated at 95 °C for 5 min to obtain the total protein. Immediately prior to electrophoresis with 10% SDS-PAGE, protein samples were transferred onto PVDF membranes (Millipore Co., Belford, MA, US). After blocking with 5% bovine serum albumin (BSA), the membranes were incubated with primary antibody overnight at 4 °C and secondary antibody at room temperature for 1 h. Following incubation with developer, the fluorescent signal was detected using a Molecular Imager ChemiDoc™ XRS Imaging System (Bio-Rad Laboratories, Hercules, CA, USA).

### 2.5. Statistical Analysis

The results have been represented as the mean ± standard deviation (SD). Significance between the two groups was analyzed by Student’s *t*-test using the SPSS 20.0 (SPSS Inc., Chicago, IL, USA) software package, and *p*-values less than 0.05 were considered to be statistically different.

## 3. Results and Discussion

### 3.1. Physicochemical Properties and Molecular Weight

In Table 1, the yield of CYP obtained from Chinese yam was 0.21 ± 0.01%, and the contents of carbohydrate, uronic acid, protein were 33.62 ± 0.08%, 34.95 ± 0.21%, and 5.21 ± 0.26%, respectively. The finding indicated that CYP was an acidic polysaccharide. The molecular weight of CYP was 20.89 kDa based on T-series dextran as the standard.

### 3.2. Monosaccharide Compositions

Monosaccharide compositions were determined by ion chromatography. The results demonstrated that CYP was composed of arabinose (Ara), galactose (Gal), glucose (Glu), mannose (Man), xylose (Xyl), rhamnose (Rha), galacturonic acid (GalA), and glucuronic acid (GluA) (Table 1). CYP mainly consisted of Gal (28.57%), GluA (11.28%), and GalA (37.59%), which indicated that Gal, Glu, and GalA might form the backbone structure of CYP. However, the result was different from the polysaccharides prepared by Huang et al. [34], which could be due to raw materials produced in different periods and different extraction methods.

### 3.3. UV and FT-IR Spectrum Analysis

**Figure 1:**
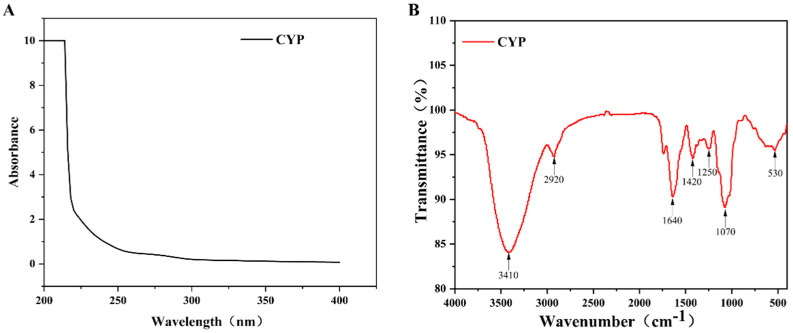
(**A**) UV-vis and FT-IR spectra of CYP (**B**) UV-vis spectra were recorded in the range of 200−400 nm. FT-IR spectra were recorded with a Nicolet 5700 FT-IR spectrometer between 400 and 4000 cm^−1^ using the KBr-disk method.

UV spectrum analysis of CYP showed that there was no absorption at 260 and 280 nm (Figure 1A), which indicated that CYP barely contained nucleic acids and proteins. This finding was consistent with the previous physicochemical-property analysis [35]. As shown in Figure 1B, a broad and strong absorption peak at 3410 cm^−1^ was attributed to the O–H stretching vibration, and a relatively weak absorption peak at 2910 cm^−1^ was assigned to the C–H stretching vibration [36]. The absorption peak at 1646 cm^−1^ might be caused by the O–H bending vibration [37]. A weak absorption band at 1420 cm^−1^ could originate from the stretching vibration of the carboxyl symmetry [38], which proved the presence of uronic acid. It could be confirmed by the results of the physicochemical properties and monosaccharide composition of CYP. The absorption peak at 1250 and 1070 might be caused by the stretching vibration of C–O [39], indicating the presence of the pyranose ring [40]. Combined with the above analysis, CYP had a clear polysaccharide characteristic.

### 3.4. SEM Analysis

**Figure 2:**
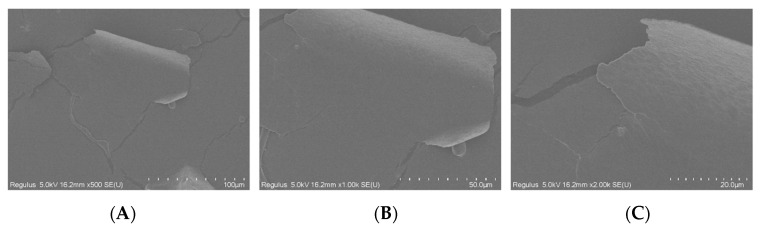
SEM micrographs showing surface microstructure of CYP ((**A**–**C**), 500×, 1000×, 2000× respectively).

Scanning electron microscopy (Figure 2) can observe the surface morphology of polysaccharides, providing direct information on their microscopic appearance. Different from most polysaccharides that have a granular shape [32], CYP consists of irregular pieces that were observed at low magnification. After the magnification of 2000 times, many polymerized polygonal pieces can be seen on the surface of turmeric polysaccharide [41], but the surface of CYP is relatively smooth. This finding may be due to the relatively small molecular weight of CYP.

### 3.5. Effect of CYP on IEC-6 Cells Viability

**Figure 3:**
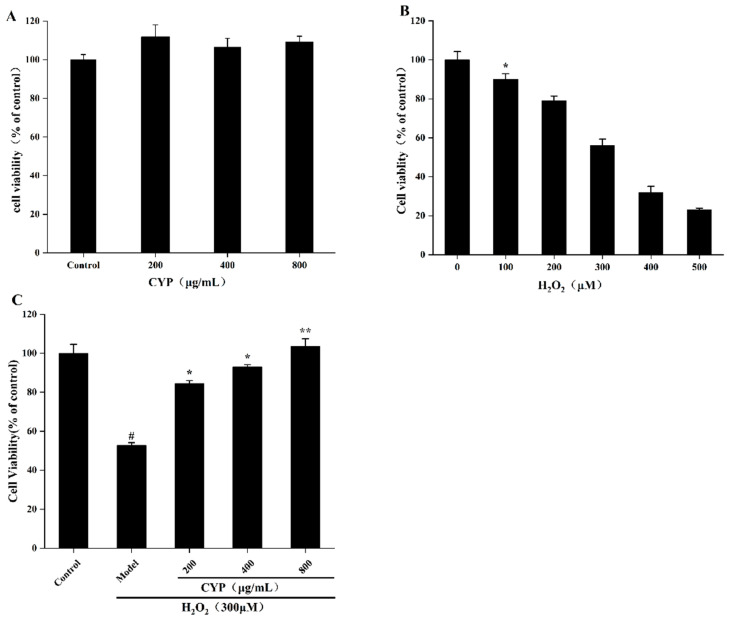
(**A**) Toxicity test of CYP on cell viability of IEC-6 cells (% of control), (**B**) Effects of H_2_O_2_ on cell viability of IEC-6 cells (% of control), (**C**) Effects of CYP on cell viability in H_2_O_2_-injured IEC-6 cells (% of control). Results shown are expressed as means ± SD (*n* = 3). # *p* < 0.05 compared with normal group, * *p* < 0.05 compared with H_2_O_2_ group alone ** *p* < 0.01 compared with H_2_O_2_ group alone.

The cell viability of different concentrations of CYP cultured with IEC-6 cells is shown in Figure 3A. Compared with the control group, IEC-6 cells co-cultured with three concentrations of CYP had comparable cell viability, indicating that CYP had no toxic effect on IEC-6 cells.

In constructing a model of cellular oxidative damage by stimulating IEC-6 cells with different concentrations of H_2_O_2_, all concentrations of H_2_O_2_ reduced the viability of IEC-6 cells in a dose-dependent manner (Figure 3B). The cell viability of the 300 µM group was 55.83% compared with the control group. Therefore, 300 µM H_2_O_2_ was used as the concentration of the model group for the subsequent experiments.

The cell viability of the model group was significantly reduced after H_2_O_2_ stimulation of the cells. The pre-protected polysaccharide group was able to effectively slow down this trend, where the cell viability of the high-concentration group was comparable to that of the control group (Figure 3C). Thus, CYP had a better protective ability to deal with the negative effects of H_2_O_2_, consistent with a previous report [42].

### 3.6. Effects of CYP on MDA and SOD in H_2_O_2_-Stimulated IEC-6 Cells

**Figure 4:**
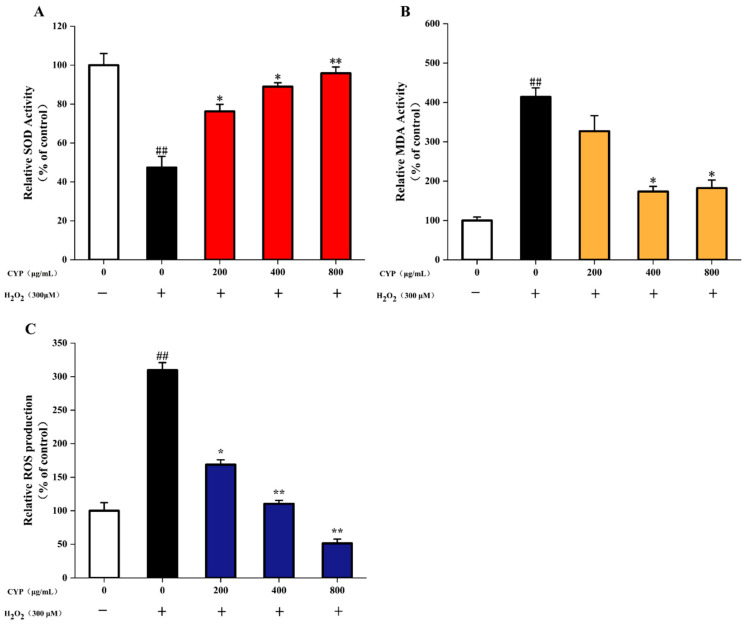
(**A**) Effects of CYP on levels of SOD in H_2_O_2_-injured IEC-6 cells, (**B**) Effects of CYP on levels of MDA in H_2_O_2_-injured IEC-6 cells, (**C**) Effects of CYP on the ROS production in H_2_O_2_-injured IEC-6 cells. Results shown are expressed as means ± SD (*n* = 3). ## *p* < 0.01 compared with normal group, * *p* < 0.05 compared with H_2_O_2_ group alone, ** *p* < 0.01 compared with H_2_O_2_ group alone.

As two important indexes for detecting the level of oxidative cell damage, SOD is an important antioxidant enzyme in organisms, which catalyzes the dismutation of superoxide anions to produce H_2_O_2_ and O_2_ [43]. MDA is a natural product of lipid oxidation in living organisms; lipid oxidation occurs when oxidative stress occurs in animal or plant cells, some fatty acids are oxidized and gradually decompose into a complex series of compounds, including MDA. Incubation of IEC-6 cells with 200 μg/mL CYP before H_2_O_2_ damage was able to increase SOD activity, and CYP showed a dose-increasing trend to enhance the activity of SOD. However, the MDA content remained high (6.04 μM). With an increase in CYP concentration to 400 and 800 μg/mL, the level of MDA decreased to 4.77 and 2.54 μM compared to control (1.46 μM) (Figure 4). Pretreatment with CYP significantly inhibited H_2_O_2_-induced oxidative damage, reduced MDA levels, and increased antioxidant enzyme activity. These results suggested the potential antioxidant capacity of CYP [44].

### 3.7. ROS Production in H_2_O_2_-Induced IEC-6 Cells

Excessive ROS production can damage cells and lead to cellular oxidative stress, which can cause the development of various diseases [6]. H_2_O_2_ significantly increased the ROS level in IEC-6 cells to 3.1-fold (Figure 4C) compared with the control group. The high dose (800 μg/mL) of CYP significantly reduced the release of intracellular ROS. Interestingly, this effect was higher when compared to control. Moreover, pretreatment of IEC-6 cells with different CYP concentrations reduced the level of ROS in a significant dose-dependent manner from 200 µg/mL to 800 μg/mL. These results were similar to those of Liao et al. [7].

### 3.8. Effect of CYP on MAPK Pathway in Oxidative Stressed IEC-6 Cells

**Figure 5:**
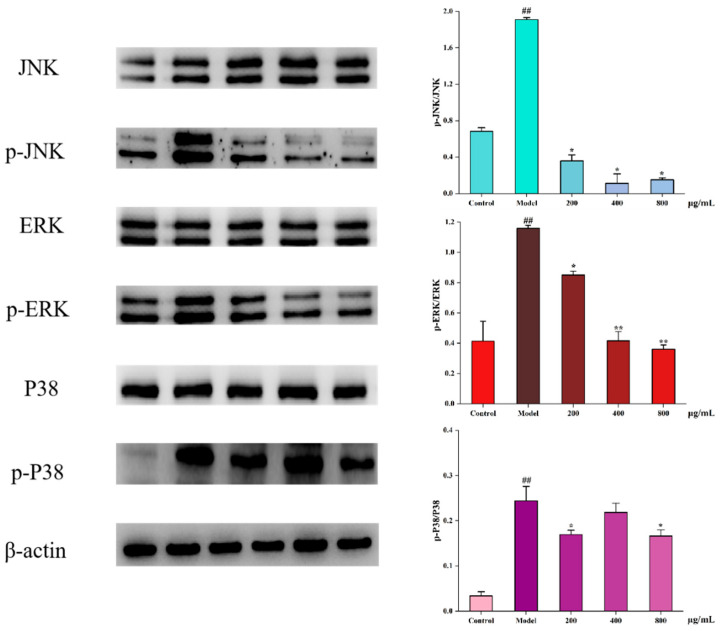
Effects of CYP on the H_2_O_2_ induced MAPK pathway of IEC-6 cells. Results shown are expressed as means ± SD (*n* = 3). ## *p* < 0.01 compared with normal group, * *p* < 0.05 compared with H_2_O_2_ group alone, ** *p* < 0.01 compared with H_2_O_2_ group alone.

MAPK is associated with oxidative stress, and the excessive activation of the MAPK pathway can lead to oxidative damage in cells [8]. Meanwhile, ROS overproduction could trigger the MAPK pathway when oxidative stress occurs in cells [45]. As observed in Figure 5, the ratios of p-JNK to JNK, p-ERK to ERK and p-P38 to P38 were significantly increased after H_2_O_2_ treatment compared to the control group. It suggested that oxidative damage to cells might be related to the MAPK pathway.

The JNK family is a key molecule in cellular signaling in response to various stressors induced by stressors and is involved in cellular responses to radiation, osmotic stress, temperature changes, and other stressors. After stimulation by H_2_O_2_, JNK protein was phosphorylated, while the level of intracellular JNK phosphorylation was significantly decreased after pretreatment with CYP.

ERK is a protein involved in the regulation of cell proliferation and differentiation. Upon oxidative stimulation, ERK phosphorylation in cells significantly increased. This phenomenon was effectively inhibited by CYP at 400 and 800 mg/mL.

P38 mediates inflammation and apoptosis. In the model group, the phosphorylation level of P38 was significantly higher than that of the control group, and the phosphorylation level of the polysaccharide group decreased relative to that of the model group. Unlike the JNK and ERK proteins, the phosphorylation level of the polysaccharide group did not decrease to that of the control group, which may be due to the fact that as an important mediator of apoptosis, the cells underwent apoptosis after oxidative damage stimulated by H_2_O_2_.

The ratios of all three proteins and their phosphorylated proteins were significantly decreased after pretreatment with CYP, which indicated that CYP might inhibit the activation of the MAPK pathway and thus protect IEC-6 cells.

## 4. Conclusions

In the present study, we isolated a polysaccharide and evaluated its protective effect against oxidative damage in cells. A water-soluble acidic polysaccharide (molecular weight = 20.89 kDa) was successfully extracted from Chinese yam. Analysis of monosaccharide composition showed that CYP primarily comprised galactose (28.57%), glucose (11.28%), and galacturonic acid (37.59%). CYP was able to increase SOD activity, inhibit MDA production, and reduce intracellular ROS production. Moreover, CYP exhibited better antioxidant activity, and it was able to alleviate the reduction of cell viability induced by oxidative stress due to H_2_O_2_ by inhibiting the activation of the MAPK pathway. It also provided a theoretical basis for the development of functional foods and clinical therapeutics against the damage caused by oxidative stress.

## Figures and Tables

**Table 1:** Chemical composition of CYP.

Sample	CYP
Yield (%)	0.21 ± 0.01
Carbohydrate (%)	33.62 ± 0.08
Uronic acid (%)	34.95 ±0.21
Protein (%)	5.21 ± 0.26
Mw (kDa)	20.89
Monosaccharide compositions	Ratio (%)
Arabinose	4.51
Galactose	28.57
Glucose	11.28
Mannose	6.77
Xylose	2.26
Rhamnose	4.89
Galacturonic acid	37.59
Glucuronic acid	4.14

Data are expressed as the mean ± SD.

## Data Availability

Not applicable.
